# Supplementary material for: Predicting malaria epidemics in Burkina Faso with machine learning
Source: PLoS One. 2021 Jun 18;16(6):e0253302. doi: 10.1371/journal.pone.0253302 (PMC8213140; doi:10.1371/journal.pone.0253302)
Supplement: S1 Appendix — (PDF) [file pone.0253302.s001.pdf]

A Accuracy tests

Here we present the accuracy tests of each confidence limit. In each case we show the fraction of mis-predicted cases as a function of predicted week. I.e. the fraction of times the one-tailed lower bound is above the ground truth, the one-tailed upper bound is below the ground truth and the ground truth lies out-side the two-tailed region. Fig 7 shows the accuracy for the one-tailed lower (left-hand figure) and one-tailed upper (right-hand figure) for the 1σ and 2σ confidence levels for the three scenarios: rising, falling and flat. The arrows on the left hand side of the Fig at week 0.5 are the mean accuracy. Fig 8 shows the results of the accuracy test for the two-tailed confidence region. We Find that the early weeks in the prediction are often more wrong than the later ones. This is because of the variance in the number of cases is greater than the

variance in the predictions. Whereas, at later times the range of possible trajectories is much greater than the intrinsic variance of the malaria cases.

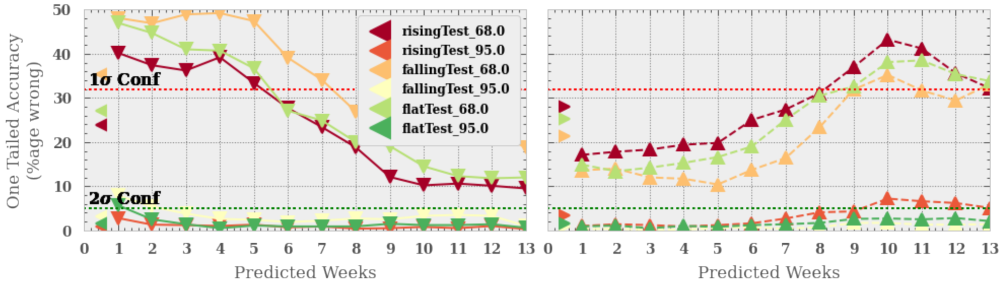

Fig 7. One-tailed accuracy tests for the lower bound (left-hand figure) and the upper bound (right-hand figure) for the three scenarios, rising, falling and flat and two confidence levels. Each line represents how often the prediction is wrong as a function of predicted week. The arrows at week 0.5 show the mean accuracy for each test over the 13 week period.

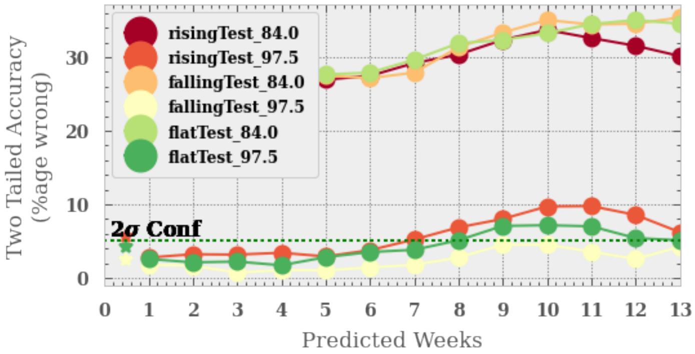

Fig 8. Two-tailed accuracy tests for three scenarios and confidence levels. Each line represents how often the ground truth lies outside the predicted region. The arrows at week 0.5 represent the mean accuracy over the 13 week period.
