# Supplementary material for: Predicting malaria epidemics in Burkina Faso with machine learning
Source: PLoS One. 2021 Jun 18;16(6):e0253302. doi: 10.1371/journal.pone.0253302 (PMC8213140; doi:10.1371/journal.pone.0253302)
Supplement: S2 Appendix — (PDF) [file pone.0253302.s002.pdf]

B Examples

Here we show ten random tests. In each case we show the number of malaria cases both predicted and true as a function of time. We show for each test the preceeding 13 weeks that we fit our model to as the solid blue line, then the predicted 1σ and 2σ confidence regions in yellow and orange resepectively. We also show the 1σ (green) and 2σ (red) lower (up-arrows) and upper (down-arrows) bounds. We stress that these upper and lower bounds are independent of each other. We also show the ground truth in the dotted line.

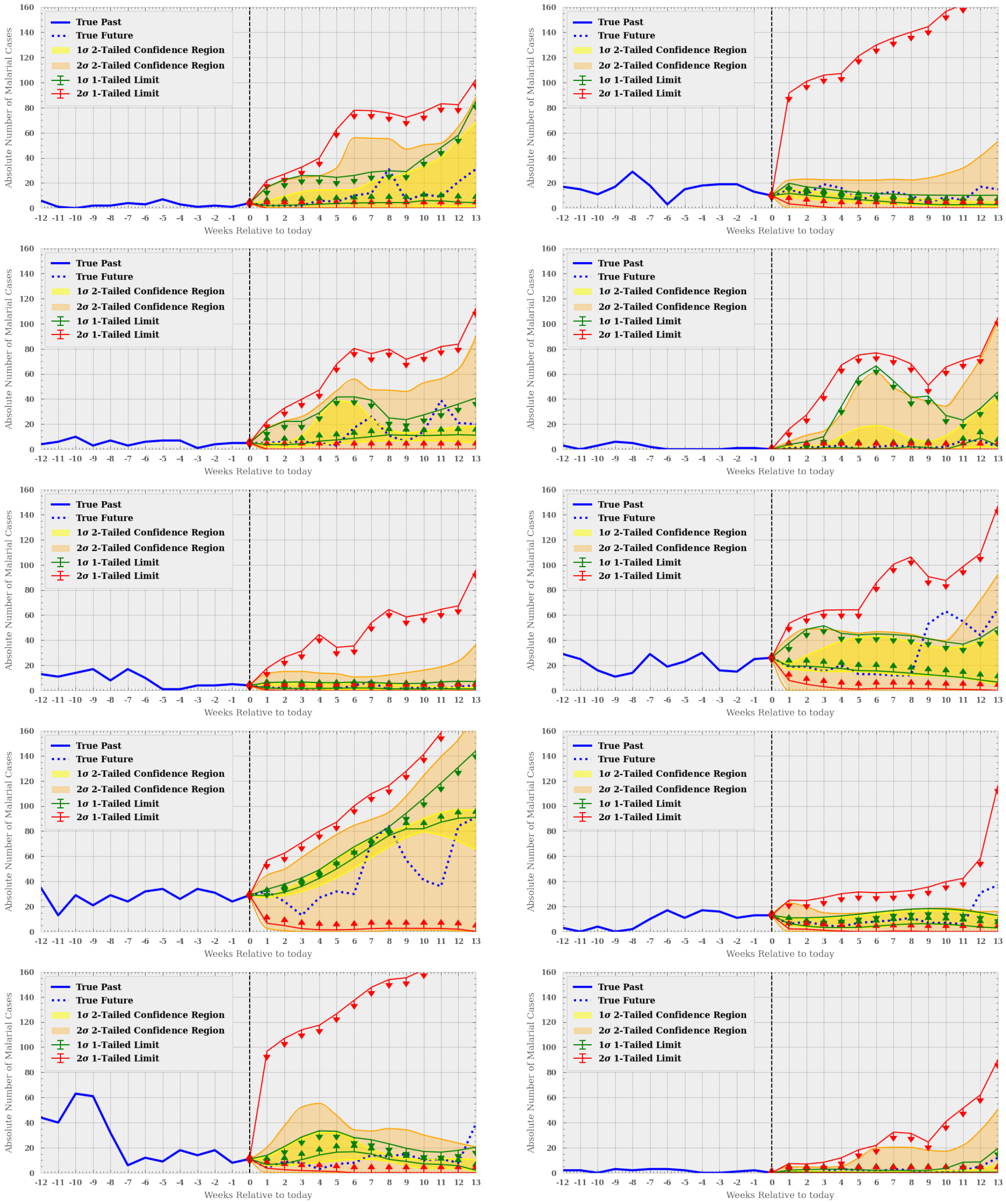

Fig 9. Ten example predictions. In each each we show the preceeding 13 weeks that we fit our model to as the solid blue line, the predicted 1σ and 2σ confidence regions in yellow and orange resepectively. We also show the 1σ (green) and 2σ (red) lower (up-arrows) and upper (down-arrows) bounds. We stress that these upper and lower bounds are independent of each other. We also show the ground truth in the dotted line.
