# Supplementary material for: Predicting malaria epidemics in Burkina Faso with machine learning
Source: PLoS One. 2021 Jun 18;16(6):e0253302. doi: 10.1371/journal.pone.0253302 (PMC8213140; doi:10.1371/journal.pone.0253302)
Supplement: Attachment — Submitted filename: ReplyToRef.pdf [file pone.0253302.s003.pdf]

Dear Editor

We would like to thank the reviewers for their comments. Below is our response to each point. We have highlighted the amended text in the manuscript in bold.

We hope with these adjustments that the paper can be accepted for publication.

Kind Regards
David Harvey, Wessel Valkenberg and Amara Amara

<u>Reviewer #1</u>

• The contributions of the authors are not clear. They have mentioned in first contribution.

We thank the referee for pointing this out. We have specifically added a section at the end of the paper.

• Several paragraphs contain trivial information and should be dropped.

We thank the referee for suggesting reducing the size of the paper. We have now significantly reduced its length without loss of clarity.

• Each section should have a summary table. If contents are too much, then add summary tables for the subsection.

We are not entirely sure we understand what the reviewer is suggesting here.

• Each section should present new information and perspective to enlighten the readers.

We have now reduced the size of the paper such that each section hopefully meets this criterion.

• Paper contributions are presented without pitching problems in recent studies. Add one paragraph before it to highlight issues in recent studies and at the end how this paper overcome those shortcomings.

We thank the referee for suggesting we reorganize the introduction. We have now re-written it.

• Improve the presentation and resolution of Fig. 3,4,5,6. It’s a very informative figure.

We thank the referee, we have improved the figures now.

• writing is good, need to check the typo errors.

We have proof read the manuscript again. We apologise if a few have slipped through the net.

• Paper is well-formatted, plz check the formatting of the reference

We thank the referee for this and have altered the formatting to be in line with the journal.

• I found some English mistakes please check them.

We have proof read the manuscript again. We apologise if a few have slipped through.

• There is lot of relevant literature missing. Please cite the below articles but not limited to:

We thank the referee for suggesting these articles. We have reviewed the introduction and added more references.

Reviewer #2:

1. The authors have to proofread the article carefully. There are many grammatical errors in the paper.

We have proof read the manuscript again. We apologise if we have missed a few.

2. There are several long sentences in the paper. Try to use simple and short sentences.

We thank the referee for pointing this out. We have tried to reduce the paper and simplify it without loss of clarity.

3. The abstract has to be rephrased.

We have now rephrased the abstract and hope it is suitable.

4. What are the limitations of the existing works that motivated the current research?

We now motivate the work more clearly at the end of “Data driven models”

5. List out the main contributions of the current work.

We thank the referee for pointing this out. We have specifically added a section at the end of the paper.

6. The related work can be summarized as a table.

Does the referee mean the author contributions? If so we have added a section now and hope that means the criteria.

7. Some of the recent and relevant works on disease prediction and machine learning such as the following can be discussed in the paper.

We thank the referee for their comments. We have now added the relevant references to the introduction.

8. Compare the current work with recent state-of-the-art.
9. Discuss about the limitations of the current work in conclusion.

We thank the referee for these important comments. Indeed including the limitations in the conclusions is vital and have therefore added this. However, comparison to state-of-the-art is difficult, because there isn’t one currently. However, we have noted a comparison with simulations.
